# Clinical utility of nucleic acid amplification testing for detection of sexually transmitted infections in women with symptomatic bacterial vaginosis and vulvovaginal candidiasis identified by conventional versus molecular diagnoses

**DOI:** 10.1128/jcm.00117-26

**Published:** 2026-08-21

**Authors:** Damon Getman, Arlene C. Seña, Ashley Nenninger, Jane R. Schwebke

**Affiliations:** 1 Hologic, Inc. 315338 , San Diego, California, USA; 2 University of North Carolina 2331, Chapel Hill, North Carolina, USA; 3 University of Alabama at Birmingham 9968, Birmingham, Alabama, USA; Children's Hospital Los Angeles, Los Angeles, California, USA

**Keywords:** bacterial vaginosis, vulvovaginal candidiasis, sexually transmitted infection, nucleic acid amplification test, *Mycoplasma genitalium*, *Trichomonas vaginalis*

## Abstract

Numerous studies describe increased rates of sexually transmitted infections (STIs) in women with symptomatic vaginitis from various causes. We present a comparative analysis of the diagnostic utility of STI nucleic acid amplification testing (NAAT) for *Trichomonas vaginalis* (TV), *Chlamydia trachomatis* (CT), *Neisseria gonorrhoeae* (NG), and *Mycoplasma genitalium* (MG) in 1,039 women with symptomatic vaginitis, including 823 women diagnosed with bacterial vaginosis (BV) and/or vulvovaginal candidiasis (VVC) by in-office health care provider (HCP) clinical assessment, conventional laboratory methods (Nugent Score/Amsel Criteria/culture), or BV- and VVC-NAAT methods. Among 508 women with a conventional laboratory BV diagnosis, significantly more STIs were detected in women with a positive BV-NAAT result (147/154, 95.5%) compared to women with HCP diagnosis of BV (134/154, 87.0%; *P* = 0.0408). For women with a culture-positive VVC diagnosis, a higher STI-NAAT positivity rate correlated with a positive VVC-NAAT result (55/59, 93.2%) compared to HCP VVC diagnosis (18/59, 30.5%), although this difference was not significant. A positive vs negative BV-NAAT result had a significantly higher relative risk for any STI infection (RR: 1.85; 95% confidence interval [CI] 1.43–2.39, *P* < 0.0001), while relative risks for STI-NAAT positive infections were not elevated significantly in VVC. Finally, STI NAAT positivity rates were higher in women with positive BV/VVC NAAT vaginitis diagnoses compared to women diagnosed with BV/VVC by conventional clinician empirical diagnosis. These results indicate combined molecular testing for STIs, and BV/VVC has improved diagnostic yield compared to conventional laboratory methods or traditional HCP assessment for these disorders.

**IMPORTANCE:**

An all-molecular (nucleic acid amplification test [NAAT]-based) approach to vaginitis and sexually transmitted infection (STI) testing of vaginal swabs from women with symptomatic vaginitis provides optimal STI case finding comparable to using STI NAATs after traditional lab-based methods for vaginitis diagnosis and better than using STI NAATs following a clinician’s assessment for vaginitis diagnosis. This study demonstrates that a combined molecular testing strategy to identify the causes of vaginitis and STIs can improve risk stratification, support guideline-aligned STI testing, help clinicians provide the most appropriate and comprehensive care, and streamline clinical and laboratory workflows using a single specimen and single test method (NAAT).

## INTRODUCTION

Sexually transmitted infections (STIs) continue to exert a significant health burden worldwide, with a reported 374 million new infections in 2020 in women ages 15–49 (1). These infections create a substantial cost to society for prevention and treatment as well as productivity time lost (2, 3). STIs are associated with serious complications, including pelvic inflammatory disease, infertility, preterm birth, cancer, congenital conditions, and increased risk of HIV acquisition and transmission (4, 5). Despite the compelling public health issues and the financial burden caused by STIs, screening for these infections is not often conducted in medical care due in part to social stigmas associated with discussing sexual health with health care providers (HCP) (6, 7). Still, other cases of STIs are not diagnosed due to nonspecific symptoms that are indistinguishable from other vaginal inflammatory and dysbiosis disorders (8).

Vaginitis is a common syndrome in women typically characterized by symptoms, including vaginal itching, burning, and irritation, dyspareunia, and abnormal vaginal odor and discharge. Major causes of vaginitis are bacterial vaginosis (BV), vulvovaginal candidiasis (VVC), and *Trichomonas vaginalis* (TV) infection. BV is a highly prevalent condition with an incidence estimated to be 21 million cases per year in the United States (U.S.) (9). Most women experience at least one episode of vaginitis in their lifetime, and it is the most common reason for women to seek medical care worldwide (10–12).

Previous studies have shown women with symptoms of vaginitis to be at significant risk for prevalent and incident infections with *Chlamydia trachomatis* (CT), *Neisseria gonorrhoeae* (NG), and *Mycoplasma genitalium* (MG) (13–18). This is especially true for women with BV, which has historically relied on an HCP assessment at the point of care to determine the presence of an abnormal vaginal discharge, elevated vaginal pH, potassium hydroxide (KOH) whiff test, and microscopic examination of fresh vaginal smear specimens for clue cells to determine Amsel Criteria. However, continued implementation of these methods is challenged by the continuing loss of availability of microscopy in clinic settings for physician diagnosis of vaginitis (19). In addition, HCP diagnosis of vaginitis based on Amsel Criteria has been shown to be less accurate than traditional laboratory-based diagnostic methods (20, 21). Laboratory-based Gram stain assessment of a vaginal sample smear for Nugent Score for BV characterization and culture for TV and *Candida* spp. are the reference standard methods, although these procedures are labor intensive and susceptible to inter-operator variability in interpretation (22, 23).

To address these increasing barriers to accurate vaginitis diagnosis and treatment, U.S. Food and Drug Administration (FDA)-cleared nucleic acid amplification tests (NAATs) for the diagnosis of BV, TV, and VVC are now available for routine testing of both self-collected and clinician-collected vaginal swab specimens from symptomatic women (20, 21, 24). These tests were developed and validated against the gold standard reference of laboratory-based consensus Nugent Score for BV and culture and Sanger sequencing for TV and *Candida* spp. (20).

Previous studies that focused on the use of NAATs for detecting STIs in women with symptomatic vaginitis were based on the diagnosis of BV and/or VVC using laboratory-based Nugent Score and culture methods, which are considered to be less sensitive than the newer NAATs for BV and VVC. We, therefore, conducted this study to investigate the clinical utility of using an all-molecular diagnostic modality for testing women with symptoms of vaginitis, in which FDA-cleared NAATs for BV and VVC were used to designate women into specific vaginitis diagnostic categories, followed by subsequent NAAT analysis for these STIs. Our results indicate that a combined molecular testing approach for BV/VVC diagnosis and for STI diagnosis provides similar or improved clinical accuracy for STI detection in women with vaginal dysbiosis compared to conventional laboratory methods or point-of-care HCP assessment for diagnosing these disorders.

## MATERIALS AND METHODS

### Study design

We conducted a secondary analysis of data collected from a prospective, multi-center, cross-sectional study of women presenting for care with symptoms of vaginitis (21). This prior study was conducted to validate the clinical performance of two FDA-cleared nucleic acid amplification tests (NAATs) for BV and VVC; details on the enrollment and consent procedures for this study are described previously (21). We used remnant specimens from this study in order to investigate the prevalence and distribution of STIs in women.

### Study population

Enrollment occurred between June and October 2018 at 21 U.S. sites consisting of clinical research centers and emergency medicine, family planning, public health, STI, and family medicine/obstetric-gynecologic clinics. Adolescent and adult women at least 14 years of age with symptoms of vaginitis (e.g., abnormal vaginal discharge, vaginal odor, genital itching or irritation, pain/discomfort during sexual intercourse or urination, edema, or erythema) were eligible for enrollment. For each participant, the collection site provided demographic and clinical data, including clinician’s diagnosis, subject-reported date of birth, sex, ethnicity, race, symptoms of STIs, pregnancy status, menstrual status, recent (within 24 h) unprotected sexual intercourse, HIV diagnosis, history of recurrent symptoms of vaginitis within 12 months, and use of feminine products within 4 weeks.

### Sample collection

Six vaginal swab samples for use in molecular testing were collected by a health care provider in the clinic from each participant during routine clinical visits using Aptima Multitest swabs (Hologic, Inc., San Diego, USA). Vaginal swabs for *Candida* spp. culture (BD BBL CultureSwab EZ; Becton, Dickinson and Company; Sparks, MD) and for Nugent Score/Amsel Criteria determination and *T. vaginalis* cultures were also obtained from each participant. The wet mount slide was evaluated for modified Amsel Criteria by the clinician within 20 min from the time of collection (25).

### Laboratory testing

For each participant, a single clinician-collected vaginal swab was smeared on a glass microscope slide to prepare the Nugent scoring slide, and then used to complete Amsel evaluation. The reference method for BV diagnosis was comprised a consensus Nugent Score and modified Amsel Criteria, if necessary. Slides were Gram stained as described previously (21) and assigned a score following independent review by three different reviewers at a single reference laboratory, blinded to each other’s interpretations. Agreement on BV interpretations (positive, negative, or intermediate) by at least two reviewers constituted consensus, and the Nugent interpretation was final. Disagreement across all three reviewers was resolved via a panel review of the same slide at a multi-headed microscope. Slides with a consensus Nugent interpretation of Intermediate were resolved using modified Amsel Criteria (≥20% clue cells together with either vaginal fluid pH greater than 4.5 or a positive whiff test (potassium hydroxide test on the swab) to determine BV status (25). This algorithm determined the laboratory-based patient infected status (PIS) for BV.

The reference method to determine PIS of VVC was laboratory-based yeast culture. For culture, a single vaginal swab from each participant was used to inoculate two different culture media (Sabouraud dextrose agar and CHROMagar *Candida*) at a single reference laboratory. The growth level on both media after 48 h was reported as follows: no colony, 1+, 2+, 3+, and 4+, with *n*+ representing the number of quadrants showing *Candida* growth. Participants with a positive culture result with either medium were categorized as positive for VVC. For trichomoniasis, the reference method comprised the combined results of culture and an FDA-cleared NAAT for *T. vaginalis*, as described previously (21). The FDA-cleared Aptima BV and Aptima CV assays were used for NAAT testing for diagnosis of BV and VVC, as previously described (21).

HCP diagnosis of vaginitis was based on the clinician’s assessment of in-clinic testing or other standard of care testing, clinical signs, patient-reported symptoms, and subject history, as previously described (21).

Archived clinician-collected vaginal swab specimens stored at −70°C were tested within validated −70°C storage times for *Neisseria gonorrhoeae* (NG), *Chlamydia trachomatis* (CT), and *Mycoplasma genitalium* (MG) using FDA-cleared transcription-mediated amplification NAATs (Aptima Combo 2 and Aptima *Mycoplasma genitalium* assays, respectively, Hologic, Inc., San Diego, USA) for detection of ribosomal RNA from each organism. All STI NAAT testing was performed at one site on the automated Panther system instrument using assay-specific software for result interpretation.

### Statistical methods

Demographic characteristics were evaluated based on the vaginosis/vaginitis laboratory diagnosis. Relative risks (RR) were computed along with the Wald confidence intervals (CI) for these estimates. Results were considered significant at the level of *α* ≤ 0.05. Fisher's exact test was used to determine significance in 2 × 2 contingency analysis for small sample sizes. Samples with inconclusive reference results and samples with invalid or missing investigational assay results were excluded from the analyses.

## RESULTS

**Fig 1:**
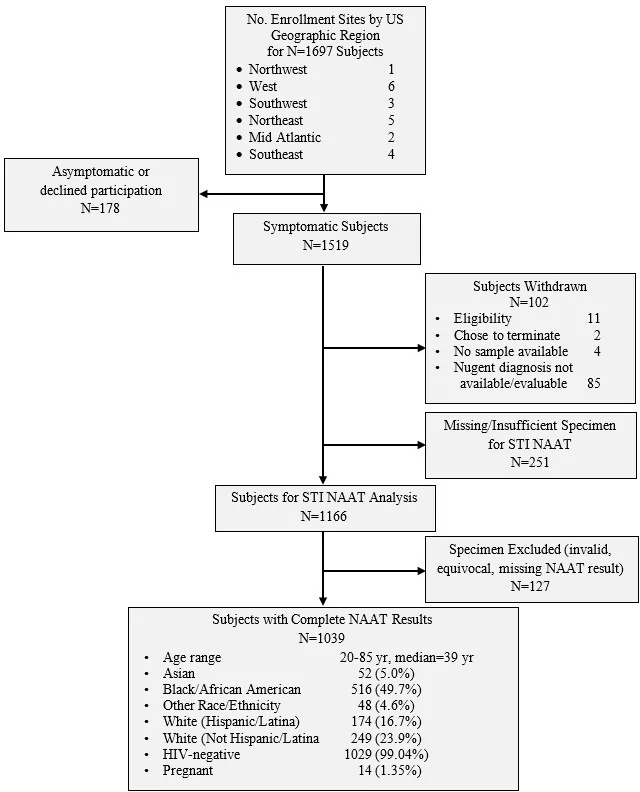
Subject and sample disposition for sexually transmitted infection nucleic acid amplification testing (NAAT) in a cohort of US women with symptomatic vaginitis.

Over the study period, 1,697 participants were recruited for enrollment at the clinical sites (Fig. 1), of which 178 were excluded since they were asymptomatic or declined participation. Among 1,519 women who were symptomatic for vaginitis and eligible for the study, 102 withdrew, and another 251 had insufficient specimens for STI NAATs. There were 1,166 participants who had STI NAATs performed, with complete test results obtained for 1,039 women. In this final cohort, the age range was 20–85 years (median 39 years, interquartile range [IQR] 16), and the majority (516, 49.7%) were Black/African American; 174 (16.7%) were White/Hispanic-Latina; 249 (23.9%) were White/Not Hispanic-Latina; and 48 (4.6%) reported as other race/ethnicity. Fourteen participants (1.35%) were pregnant, and most (1,029, 99.04%) were HIV-negative.

Table 1 shows a comparison of NAAT detection rates for *T. vaginalis* (TV), *C. trachomatis* (CT), *N. gonorrhoeae* (NG), and *M. genitalium* (MG) in women with symptomatic vaginitis diagnosed by either HCP clinical assessment or NAATs for BV and VVC (*Candida* species, Cspp.). There were 508 women with a laboratory-based patient infected status (PIS) designation of BV. Compared to this BV PIS as reference, BV NAAT detected 483 (95.1%) cases of BV compared to 431 (84.8%) cases of BV detected by HCP clinical assessment. There were 154 women with STIs detected in this BV PIS-positive cohort, with a high prevalence of TV (18.9%, 96/508) and MG (12.6%, 64/508) detected. Among BV PIS-positive participants with any STIs (*n* = 154), 147 (95.5%) were also BV NAAT-positive compared to 134 (87.0%) who were BV-positive by HCP assessment. BV NAAT diagnosis was significantly more common (*P* = 0.0408) among women with any STI compared to HCP BV diagnosis; however, detection rates for individual STIs were not significantly different between NAAT and HCP diagnosis rates for BV.

**TABLE 1:** Comparison of sexually transmitted infection nucleic acid amplification testing detection rates in women with vaginitis diagnosis from NAAT or health care provider vaginitis diagnosis

Patient infected status^a^	Diagnostic method(s)	No. (%)	STI NAAT results, no. positive (%)				
			*T. vaginalis*	*C. trachomatis*	*N. gonorrheoae*	*M. genitalium*	Any STI
Bacterial vaginosis		508 (100)	96 (100)	18 (100)	6 (100)	64 (100)	154 (100)
	BV NAAT+	483 (95.1)	89 (92.7)	18 (100)	6 (100)	62 (96.9)	147 (95.5)
	HCP BV+	431 (84.8)	79 (82.3)	15 (83.3)	5 (83.3)	56 (87.5)	134 (87.0)
	BV NAAT+/HCP BV+	416 (81.9)	76 (79.2)	15 (83.3)	5 (83.3)	55 (85.9)	131 (85.1)
	BV NAAT+/HCP BV−	67 (13.2)	13 (13.5)	3 (16.7)	1 (16.7)	7 (10.94)	16 (10.4)
	BV NAAT−/HCP BV+	15 (2.95)	4 (4.17)	0 (0)	0 (0)	1 (1.56)	4 (2.6)
	BV NAAT−/HCP BV−	10 (1.97)	3 (3.12)	0 (0)	0 (0)	1 (1.56)	3 (1.95)
	Fisher’s exact test		*P* = 0.0881	*P* = 1.000	*P* = 1.000	*P* = 0.2361	***P*** **= 0.0408**
Vulvovaginal candidiasis		315 (100)	32 (100)	6 (100)	4 (100)	30 (100)	59 (100)
	Cspp NAAT+	297 (94.3)	31 (96.9)	5 (83.3)	4 (100)	26 (86.7)	55 (93.2)
	HCP Cspp+	141 (44.8)	7 (22.6)	3 (50)	1 (25)	10 (33.3)	18 (30.5)
	Cspp NAAT+/HCP Cspp+	137 (43.5)	7 (21.9)	3 (50)	1 (25)	10 (33.3)	18 (30.5)
	Cspp NAAT+/HCP Cspp−	160 (50.8)	24 (75)	2 (33.3)	3 (75)	16 (53.3)	37 (62.7)
	Cspp NAAT−/HCP Cspp+	4 (1.27)	0 (0)	0 (0)	0 (0)	0 (0)	0 (0)
	Cspp NAAT−/HCP Cspp-	14 (4.44)	1 (3.22)	1 (16.7)	0 (0)	4 (13.3)	4 (6.8)
	Fisher’s exact test		*P* = 1.000	*P* = 1.000	*P* = 1.000	*P* = 0.272	*P* = 0.3027

^a^ Patient infected status determined by consensus Nugent Score plus Amsel Criteria for BV, dual-media culture for VVC. Cspp, *Candida* species; HCP, health care provider; +, positive; −, negative. Bold values indicate significant comparisons.

For the 315 women with a laboratory-based PIS of VVC, *Candida* species (Cspp) NAAT detected 297 (94.3%) cases, while HCP assessment detected 141 (44.8%). There were 59 STIs detected in this VVC PIS-positive cohort, of which 55 (93.2%) were also Cspp NAAT-positive, while only 18 (30.5%) were Cspp-positive by HCP assessment. However, STI NAAT detection rates were not significantly different between Cspp NAAT and HCP Cspp diagnoses, either for each individual STI or for any STI categories.

Table 2 presents a relative risk analysis for detection of STIs following vaginitis NAAT analysis of clinician-collected vaginal swab specimens. Among BV NAAT-positive vs. BV NAAT-negative specimens, relative risks were significantly elevated for TV infection (RR: 1.925 (1.3754–2.6943, *P* = 0.0001), CT infection (RR: 3.4521 (1.2985–9.1773, *P* = 0.013), and MG infection (RR: 2.2148 (1.4285–3.434, *P* = 0.0004). Relative risk (RR) for NG was elevated in BV NAAT-positive vs. -negative, but this difference was not significant (RR: 1.8529 (0.4659–7.3689, *P* = 0.3812). Adjusting for Cspp co-infection, elevated relative risks for STIs were only observed in specimens categorized as BV NAAT-positive/Cspp NAAT-negative: TV infection RR: 1.8954 (1.2708–2.6943, *P* = 0.0017); CT infection RR: 3.0236 (1.0143–9.0294, *P* = 0.0471); and MG infection RR: 2.9988 (1.6172–5.5607, *P* = 0.0005). A positive Cspp NAAT result in the absence of BV did not correlate with a significant increase in relative risks for any STI NAAT diagnostic category.

**TABLE 2:** Relative risks for detection of sexually transmitted infection following vaginitis nucleic acid amplification testing of clinician-collected vaginal swab specimens from 1039 women with symptomatic vaginitis^*a*^^,*b*^

Diagnostic method	No.	STI NAAT results, no. (%)					
		*T. vaginalis*	*C. trachomatis*	*N. gonorrhoeae*	*M. genitalium*	Any STI	
BV NAAT							
Positive	538	99 (18.4)	19 (3.53)	6 (1.1)	66 (12.23)	161 (29.92)	
Negative	501	44 (8.78)	5 (0.99)	3 (0.59)	26 (5.19)	71 (14.17)	
RR (95% CI)		**1.92 (1.37–2.69)** ***P*** **= 0.0001**	**3.45 (1.29–9.17)** ***P*** **= 0.0130**	1.85 (0.46–7.36) *P* = 0.3812	**2.21 (1.4285–3.434)** ***P*** **= 0.0004**	**1.86 (1.44–2.39)** ***P*** **< 0.0001**	
Cspp NAAT							
Positive	337	38 (11.27)	5 (1.48)	5 (1.48)	33 (9.8)	66 (19.58)	
Negative	702	105 (14.9)	19 (2.7)	4 (0.57)	59 (8.4)	166 (23.65)	
RR (95% CI)		0.7788 (0.5487–1.1055) *P* = 0.1619	0.5548 (0.2089–1.4733) *P* = 0.2371	2.5804 (0.6973–9.5486) *P* = 0.1556	1.1504 (0.7653–1.7292) *P* = 0.5004	0.8563 (0.6606–1.1102) *P* = 0.2417	
BV NAAT−/Cspp NAAT− (reference)	318	30 (9.43)	4 (1.26)	0 (0)	12 (3.77)	44 (13.84)	
BV NAAT+/Cspp NAAT−	384	75 (19.53)	15 (3.91)	4 (1.0)	47 (12.23)	122 (31.77)	
RR (95% CI)		**1.89 (1.27–2.83)** ***P*** **= 0.0017**	**3.024 (1.01–9.03)** ***P*** **= 0.0471**	7.38 (0.39–136.576) *P* = 0.1794	**2.99 (1.62–5.56)** ***P*** **= 0.0005**	**1.98 (1.44–2.72)** ***P*** **< 0.0001**	
BV NAAT−/Cspp NAAT+	183	14 (7.65)	1 (0.55)	3 (1.64)	14 (7.65)	27 (14.75)	
RR (95% CI)		0.82 (0.44–1.52) *P* = 0.5348	0.4375 (0.049–3.88) *P* = 0.4581	11.94 (0.62–229.92) *P* = 0.1003	1.95 (0.99–4.14) *P* = 0.0802	1.06 (0.68–1.66) *P* = 0.8058	
BV NAAT+/Cspp NAAT+	154	24 (15.58)	4 (2.6)	2 (1.3)	19 (12.33)	39 (25.32)	
RR (95% CI)		1.56 (0.94–2.59) *P* = 0.0829	2.038 (0.52–8.04) *P* = 0.3094	10.6 (0.49–210.35) *P* = 0.1337	**3.02 (1.50–6.07)** ***P*** **= 0.0019**	**1.66 (1.12–2.47)** ***P*** **= 0.0115**	

^*a*^ Values are *N* (%), unless otherwise specified.

^*b*^ Bold values indicate significant comparisons. BV, bacterial vaginosis; Cspp, *Candida* species; RR, relative risk; NAAT, nucleic acid amplification test.

**Fig 2:**
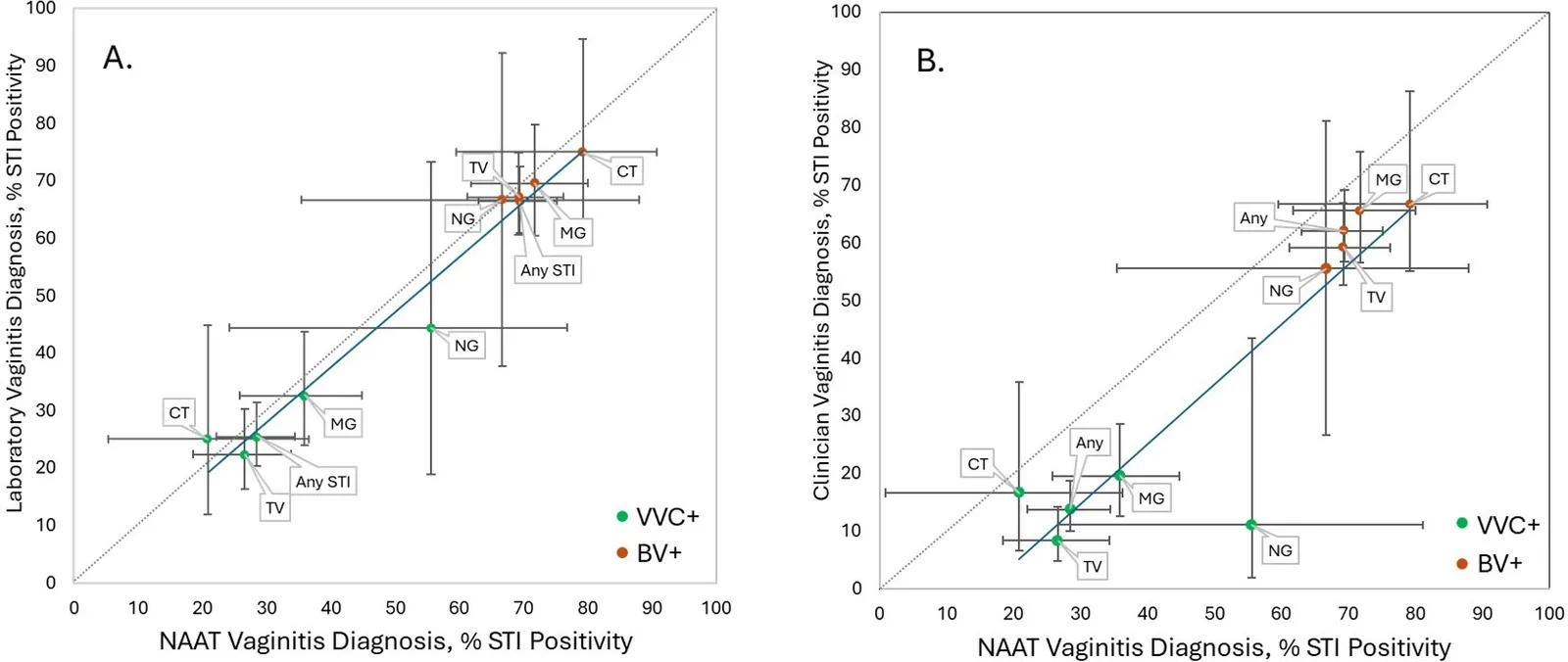
Accuracy for detection of sexually transmitted infections in specimens collected from women with symptomatic bacterial vaginosis or vulvovaginal candidiasis. STI positivity rates following vaginitis nucleic acid amplification test (NAAT) diagnosis compared to (**A**) conventional laboratory vaginitis diagnostic methods (consensus Nugent Score plus modified Amsel Criteria, or Candida culture, respectively) or to (**B**) clinician vaginitis diagnosis at point of care. CT, *Chlamydia trachomatis*; TV, *Trichomonas vaginalis*; NG, *Neisseria gonorrhoeae*; MG, *Mycoplasma genitalium*. BV+ (red) = BV positive by lab-based reference method (*N* = 508); VVC+ (green) = VVC positive by lab-based reference method (*N* = 315).

A graphical depiction of STI detection rates in specimens with either a laboratory diagnosis or a health care provider diagnosis of VVC or BV compared to a NAAT diagnosis of VVC and BV is shown in Fig. 2. Positivity rates for TV, CT, NG, and MG detection by NAAT were similar in specimens diagnosed with either laboratory-based methods for BV and Cspp infection or with NAAT-based methods for these conditions. On average, NAAT positivity rates for STI detection were 2.8% higher overall among participants with BV NAAT diagnosis compared to laboratory BV diagnosis (data in Table S1), although as shown in Fig. 2, the confidence intervals for CT and NG detection rate estimates were large. Similarly, for VVC diagnosis, NAAT STI positivity rates were 6.2% higher using Cspp NAAT diagnosis compared to laboratory Cspp diagnosis, although again with large confidence intervals for CT and NG detection rate estimates. For the comparison of NAAT STI positivity rates in specimens diagnosed with NAAT-based methods for BV and Cspp infection or by HCP diagnosis of BV and VVC infection at the point of care, on average, NAAT STI positivity rates were 13.9% higher using BV NAAT diagnosis compared to HCP BV diagnosis, while NAAT STI positivity rates were 53.5% higher using Cspp NAAT diagnosis compared to HCP VVC (Cspp) diagnosis, although, again, the confidence intervals for CT and NG STI detection rate estimates in both analyses were large, indicating the statistical non-significance of this difference.

## DISCUSSION

This study compared STI detection via NAATs in specimens collected from women diagnosed with vaginitis (BV and/or VVC) using either conventional laboratory-based methods, BV/VVC nucleic acid amplification tests, or in-clinic HCP assessment. We found similar rates of STI detection in specimens following a gold standard (consensus Nugent scoring with Amsel Criteria to referee intermediate scores) conventional laboratory-based diagnosis for vaginitis compared to a NAAT-based diagnosis of vaginitis. Women in either of these categories had significantly higher STI detection rates than STIs diagnosed in women categorized as having vaginitis using HCP in-clinic assessment perhaps due to the lower sensitivity of clinic-based empirical diagnosis methods for identifying women with BV. For *M. genitalium*, current CDC guidelines call for testing in women with recurrent cervicitis and PID, but not vaginitis. The twofold increase in MG infection in BV-positive vs. BV-negative women found in this study suggests that a reappraisal of the risks of MG infection in relation to vaginal dysbiosis may be warranted.

Using NAATs for testing urogenital specimens for STIs has been the standard of care for over 20 years, having supplanted long-standing culture methods and HCP physical diagnosis to provide a faster and more accurate method for diagnosis (26). Employment of the NAAT technology for the diagnosis of vaginitis is a more recent development, with the first FDA-cleared tests becoming available in 2017 and 2019 (20, 21). Several factors contributed to the advent of molecular testing for vaginitis, including a shortage of qualified laboratory personnel available for performing gram stain-based diagnosis for BV, increasing labor costs for culture-based *Candida* diagnosis, and the declining availability and use of microscopy in physician-operated laboratories, which decreased over 35% between 2010 and 2024 (3).

Previous studies have reported STIs to be highly associated with symptomatic BV (13, 14, 16, 18, 27). In particular, TV and MG have been shown to be significantly associated with a diagnosis of symptomatic BV, even after adjusting for VVC diagnosis and CT and NG infection (18, 28, 29). The clinical study underlying the analysis presented here was the first to include MG as part of the STI assessment in women diagnosed with vaginitis by various methods. Given the close association of STIs with symptomatic vaginitis, testing for both conditions is warranted either as concurrent testing in all women with symptoms of vaginitis or as reflex STI testing in women with a positive BV NAAT result. Either scenario can be accomplished by using a single self-collected or clinician-collected vaginal swab sample, as noted in previous studies (18, 25).

The lack of a significant association between VVC and increased STI risk is noteworthy. A positive *Candida* NAAT result, in the absence of BV, did not correlate with statistically significant higher risk for any STI tested. This supports previous findings that, unlike BV, VVC is not a marker for altered vaginal ecology that predisposes to STI acquisition (18). This distinction is clinically relevant, as it suggests that women presenting with candidiasis—when not co-infected with BV—may not require enhanced STI screening beyond standard recommendations.

Strengths of this study include the use of FDA-cleared NAATs for STI detection and for diagnosis of BV and VVC, as well as the use of clinical specimens obtained from a broad geographic and demographic sampling of women in the United States seeking care for symptomatic vaginitis. Also important are the use of consensus Gram stain scoring for the laboratory BV diagnosis combined with the employment of standardized Amsel Criteria to adjudicate the diagnosis of BV in specimens with intermediate (4 to 6) Nugent Scores, as well as the use of a combination of two culture methods plus PCR bidirectional sequencing for diagnosing VVC.

Limitations of the study include the relatively small number of CT and NG infections detected in the study cohort, which results in large confidence intervals for comparison estimates and which, therefore, confounds the analyses performed to determine the significance of association of these STIs with specific causes of vaginitis.

In conclusion, we found that an all-molecular approach to the diagnostic evaluation of vaginal swab specimens collected from women with symptomatic vaginitis provided a degree of clinical accuracy for STI detection on par with employing STI NAATs on specimens categorized using gold-standard conventional laboratory methods for vaginitis diagnosis. The availability of clinically accurate molecular tests for both STI and vaginitis diagnosis running on high-throughput automated analyzer systems should increase the ability of care providers to provide consistent and accurate non-empirical diagnostic information for guiding the management of women suffering from vaginal dysbioses, as well as provide operational efficiencies across the clinic and the laboratory with a single specimen collection, no additional HCP assessments required, and a single laboratory method (NAAT) to cover all necessary testing.

## SUPPLEMENTAL MATERIAL

Table S1 jcm.00117-26-s0001.docx Figure 2 data presented as a table.

ASM does not own the copyrights to Supplemental Material that may be linked to, or accessed through, an article. The authors have granted ASM a non-exclusive, world-wide license to publish the Supplemental Material files. Please contact the corresponding author directly for reuse.
