# Supplementary material for: Clinical utility of nucleic acid amplification testing for detection of sexually transmitted infections in women with symptomatic bacterial vaginosis and vulvovaginal candidiasis identified by conventional versus molecular diagnoses
Source: J Clin Microbiol. 2026 Aug 21;64(9):e00117-26. doi: 10.1128/jcm.00117-26 (PMC13556187; doi:10.1128/jcm.00117-26)
Supplement: Supplementary file 1 [file jcm.00117-26-s0001.docx]

**Supplemental Table 1.** Figure 2 data presented in table format.

| Figure 2A data | **Vaginitis Category** | **NAAT STI Diagnosis** | **NAAT STI Diagnosis, N** | **Vaginitis diagnosis by NAAT, n** | **Vaginitis diagnosis by NAAT, %** | **Vaginitis laboratory diagnosis, n** | **Vaginitis laboratory diagnosis, %** | **Absolute difference** | **% Difference** | **Average % diff** |
| --- | --- | --- | --- | --- | --- | --- | --- | --- | --- | --- |
|  | BV | TV | 143 | 99 | 69.23 | 96 | 67.13 | 2.10 | 3.03 | 2.83 |
|  |  | CT | 24 | 19 | 79.17 | 18 | 75.00 | 4.17 | 5.26 |  |
|  |  | NG | 9 | 6 | 66.67 | 6 | 66.67 | 0.00 | 0.00 |  |
|  |  | MG | 92 | 66 | 71.74 | 64 | 69.57 | 2.17 | 3.03 |  |
|  |  | Any | 232 | 161 | 69.40 | 154 | 66.38 | 3.02 | 4.35 |  |
|  | Cspp | TV | 143 | 38 | 26.57 | 32 | 22.38 | 4.20 | 15.79 | 6.22 |
|  |  | CT | 24 | 5 | 20.83 | 6 | 25.00 | -4.17 | -20.00 |  |
|  |  | NG | 9 | 5 | 55.56 | 4 | 44.44 | 11.11 | 20.00 |  |
|  |  | MG | 92 | 33 | 35.87 | 30 | 32.61 | 3.26 | 9.09 |  |
|  |  | Any | 232 | 66 | 28.45 | 59 | 25.43 | 3.02 | 10.61 |  |
|  |  |  |  |  |  |  |  |  |  |  |
| Figure 2B data | **Vaginitis Category** | **NAAT STI Diagnosis** | **NAAT STI Diagnosis, N** | **Vaginitis diagnosis by NAAT, n** | **Vaginitis diagnosis by NAAT, %** | **Vaginitis diagnosis by HCP, n** | **Vaginitis diagnosis by HCP, %** | **Absolute difference** | **% Difference** | **Average % diff** |
|  | BV | TV | 143 | 99 | 69.23 | 84 | 58.7 | 10.49 | 15.15 | 13.8 |
|  |  | CT | 24 | 19 | 79.17 | 16 | 66.7 | 12.50 | 15.79 |  |
|  |  | NG | 9 | 6 | 66.67 | 5 | 55.6 | 11.11 | 16.67 |  |
|  |  | MG | 92 | 66 | 71.74 | 61 | 66.3 | 5.43 | 7.58 |  |
|  |  | Any | 232 | 161 | 69.40 | 144 | 62.1 | 7.33 | 10.56 |  |
|  | Cspp | TV | 143 | 38 | 26.57 | 12 | 8.4 | 18.18 | 68.42 | 53.5 |
|  |  | CT | 24 | 5 | 20.83 | 4 | 16.7 | 4.17 | 20.00 |  |
|  |  | NG | 9 | 5 | 55.56 | 1 | 11.1 | 44.44 | 80.00 |  |
|  |  | MG | 92 | 33 | 35.87 | 18 | 19.6 | 16.30 | 45.45 |  |
|  |  | Any | 232 | 66 | 28.45 | 32 | 13.8 | 14.66 | 51.52 |  |
